# Modeling and Implementation of Cattle/Beef Supply Chain Traceability Using a Distributed RFID-Based Framework in China

**DOI:** 10.1371/journal.pone.0139558

**Published:** 2015-10-02

**Authors:** Wanjie Liang, Jing Cao, Yan Fan, Kefeng Zhu, Qiwei Dai

**Affiliations:** Institute of Agricultural Economics and Information, Jiangsu Academy of Agricultural Sciences, Nanjing, China; University of Florida, UNITED STATES

## Abstract

In recent years, traceability systems have been developed as effective tools for improving the transparency of supply chains, thereby guaranteeing the quality and safety of food products. In this study, we proposed a cattle/beef supply chain traceability model and a traceability system based on radio frequency identification (RFID) technology and the EPCglobal network. First of all, the transformations of traceability units were defined and analyzed throughout the cattle/beef chain. Secondly, we described the internal and external traceability information acquisition, transformation, and transmission processes throughout the beef supply chain in detail, and explained a methodology for modeling traceability information using the electronic product code information service (EPCIS) framework. Then, the traceability system was implemented based on Fosstrak and FreePastry software packages, and animal ear tag code and electronic product code (EPC) were employed to identify traceability units. Finally, a cattle/beef supply chain included breeding business, slaughter and processing business, distribution business and sales outlet was used as a case study to evaluate the beef supply chain traceability system. The results demonstrated that the major advantages of the traceability system are the effective sharing of information among business and the gapless traceability of the cattle/beef supply chain.

## Introduction

In recent years, beef has become an important food in China because of its perceived nutritional value among consumers, thus concerns about beef quality and safety have increased greatly. Traceability systems have been used as effective tools for guaranteeing the quality and safety of food products by improving the supply chain transparency[1–3]. Traceability can be useful for optimizing production planning and scheduling, and for improving company coordination in supply chain[4–6]. In addition, traceability can also be used as part of a competitive strategy[7]. However, food traceability is lacking because of the traceability information related to food products and production processes is often lost within companies, as well as between companies in supply chain[8–10], thus more detailed studies of each step in the supply chain are needed to better document each process[11–14]. In addition, due to the lack of standardized communication between different traceability systems, it is difficult to realize information sharing[15,16]. Researchers have done a lot of work in the communication standardized and the ability of monitoring of the whole supply chain. For example, Pizzuti et al.(2014) proposed a solution to seamless interoperability of disparate systems based on Ontology-based approaches, and Pizzuti et al.(2015) developed a Global Track and Trace system for food supply chain which involves the analysis and modelling of products flow and information flow[17,18]. Monitoring the flow of food products, their quality, and the process parameters throughout the production process and linking them to each transition in the states of these products is an effective approach for ensuring product safety and traceability[19]. In order to achieve a fully traceable supply chain, it is important to develop systems that facilitate internal traceability and external supply chain traceability[20].

## State of the Art

This section provided a brief overview of the state of the art about traceability systems based on a RFID-based framework proposed in literature. An extensive analysis of academic and trade literature of supply chain traceability systems, technologies and architectures has been carried out by Musa et al.(2014) [21]. The authors described the radio frequency identification (RFID) technology and some RFID-based system architectures in detail. They illuminated RFID deployment architectures of supply chain traceability system, which include the EPCglobal network, the SaviTrak system, the microsoft BizTalk RFID and the Sun Java System RFID, and discussed the properties of this architectures. The compared results show that only the EPCglobal network uses open, worldwide standards that are supported by GS1 and the EPCglobal network’s standards are essentially RFID-centric, in which case the device layer is rather simple.

Radio frequency identification technology, as an automatic identification and data capture technology, is considered to be the core enabling technology that facilitates traceability and their implementations are increasing at a fast rate[22]. With the implementation of RFID technology, food traceability systems can become more reliable and efficient since RFID enables a higher reading rate than traditional barcodes[23]. RFID has been widely applied in agro-food logistics and supply chain management processes for food traceability, and quality oriented tracking and tracing systems since some years ago[24–27]. Abad et al.(2009) proposed a solution for real-time traceability and cold chain monitoring for food applications based on RFID. The heart of the RFID tracing system is a smart RFID tag which integrated light, temperature and humidity sensors, microcontroller, memory chip, low power electronics and an antenna [28]. Qian et al.(2012) developed a Wheat Flour Milling Traceability System (WFMTS). In the system, the Quick Response Code (QR Code) were used for identifying the processing information of small packages of wheat flour, and RFID tags were affixed to the storage bins to record logistics information[29]. Piramuthu et al.(2013) analyzed the effect of selecting a traceability level and identification technology for a perishable food supply network, and claimed that item-level or even case-level traceability information generated using RFID technology might be more appropriate [30]. Barge et al.(2014) proposed a complete item-level traceability system for high-value, pressed, long-ripened cheese based on RFID. The authors experimented with different techniques for fixing tags to the cheese and presented solutions for automatic identification adapted to handling procedures as implemented in a dairy factory[31].

There are many RFID-based frameworks of traceability system, i.e the EPCglobal network, the SaviTrak system, the Microsoft BizTalk, and the Sun Java System RFID software architecture. However, the EPCglobal network framework is increasingly recognized as the de facto standard for RFID implementation in order to support open supply chains[32,33]. Recently, the development of traceability systems in the food supply chain based on the EPCglobal network has interested many authors. In order to automatically obtain the supply network associated to a specific product, Muñoz-Gea et al.(2010) developed a Discovery Services (DS) prototype based on Distributed Hash Tables (DHT) and an access control service, which is the main component of the EPCglobal network[34]. Nam and Yeom (2011) proposed a business-aware framework which provides high-level events, middleware, and components for developing RFID business applications cost-effectively on the EPCglobal network[35]. In order to improve the efficiency of traceability information retrieval, Ko et al.(2011) and Kang et al.(2013) focused on the development of product search algorithm and generic traceability services based on EPCglobal network[36,37]. Model and integrated traceability system based on the EPCglobal network have been proposed for specific sectors, such as fresh vegetables and aquaculture[38,39].

The analysis of the state of the art about traceability systems based on a RFID-based framework showed that different traceability systems based on RFID framework have been proposed in literature. In the research field of the food supply chain based on the EPCglobal network, several researchers paid attention to the study the product search algorithm and generic system services, and others modeled and implemented traceability system for specific sectors. Moreover, according to Karlsen et al. (2013), there’s not a sound common theoretical framework with respect to implementation of food traceability, and further theoretical developments on implementations of food traceability are needed[40].

## Model of Traceability Information

### 3.1 Traceability unit model

#### 3.1.1 Traceability unit transformation throughout the beef supply chain

A traceability unit is an object used for tracking and tracing throughout the food supply chain, which comprises an identification code and traceability information. Based on the business flow of cattle/beef supply chain and the method proposed by Feng et al.(2013)[41], a model was proposed for describing the traceability unit transformation process. In the model, the EPCIS event was introduced for expressing critical event and the relationship between parent traceability unit and child traceability units in the cattle/beef supply chain. The model was formalized as:

$$P(U)=\{ID(U),Event(U),Infor(U)\}\;(U=\{U_{1},U_{2},\cdots ,U_{n}\})$$ (1)

where *U* is a set of traceability units, *U*_*i*_(*i* = 1,2,⋯*n*) is the traceability unit identified in the beef supply chain, and *ID*(*U*) is the unique identification code of the traceability unit. The EPCIS event information for a traceability unit is described by *Event*(*U*). *Infor*(*U*) denotes the critical traceability information.

During the breeding process, the traceability unit is a cow, which is described as *U*_1_ and the corresponding data model is *P*(*U*_1_). During the slaughter and processing stage, the traceability unit transformation process is complex. Before the cattle are slaughtered, the traceability unit is still a cow, which is described as *U*_2_, and the corresponding data model is *P*(*U*_2_). Next, the cattle carcass is split into two halves and acid decomposition treatment is carried out on the dyad. In this process, the traceability unit transforms into dyad, which is described as *U*_3_, and the corresponding data model is *P*(*U*_3_). During the segmentation process, according to the business needs and the position of the cattle, the dyad is segmented into different beef products. After segmentation, the traceability unit is transformed into beef, which is described as *U*_4_, and the corresponding data model is *P*(*U*_4_). After vacuum packing, beefs are packed into different product boxes based on the product type. Next, the packaged beef products are placed in cold storage. After boxing, the traceability unit transforms into a packaged beef product, which is described as *U*_5_, and the corresponding data model is *P*(*U*_5_).

#### 3.1.2 Method for the identification of traceability unit

In the proposed method, RFID technology and barcode technology are employed as the identification method and the carrier for traceability information, respectively. Ear tag codes and EPCs are used to establish the traceability unit coding system. The identification codes for cattle are encoded according to Decree No. 67 issued by the Ministry of Agriculture of the People’s Republic of China[42]. The code comprises 15 digits, where the first digit represents the type of livestock, i.e., “2” represents cattle, the next six digits refer to the administrative region code of the county (city) in terms of GB T2260_1999[43], and the final eight digits are the sequence order numbers. When the cattle are slaughtered and divided into dyads, the operator generates two new codes, which are encoded by increasing one digit after the ear tag code as the identification code for dyads. During the segmentation process, the operator generates some new codes, which are encoded by adding two digits after the identification code of the dyad as the identification code for beef products. According to the EPC standard developed by Auto ID and sponsored by MIT (Massachusetts Institute of Technology) and EPCglobal[44], a unique serialized global trade item number (SGTIN) is assigned to a beef product. Table 1 showed an example of traceability unit transformation in the cattle/beef supply chain.

**Table 1:** Instance of traceability unit transformation in cattle/beef supply chain.

	Identification code	Location	Object
*P*(*U*_1_)	232011600000005	Breeding farm	Entire cattle
*P*(*U*_2_)	232011600000005	Slaughter and processing plant	Entire cattle
*P*(*U*_3_)	2320116000000051	Slaughter workshop	Dyad
*P*(*U*_4_)	232011600000005101	Vacuum packaging workshop	Beef
*P*(*U*_5_)	(01)80614142125437(21)50	Packaging workshop, Distribution Enterprise and Sales Enterprise	Packaged beef product

### 3.2 Internal and external traceability information acquisition and transmission

In this study, EPCIS Events were used for modeling the traceability information of beef supply chain. EPCIS is an EPCglobal standard designed to enable EPC-related data sharing within and across enterprises. EPCIS mainly deals with event data which is created in the process of carrying out business processes and captured through the EPCIS Capture Interface. The EPCIS events cover normal logistic and stock control processes by the use of the Event classes: ObjectEvent, AggregationEvent, QuantityEvent and TransactionEvent. The basic chain traceability requirements with respect to managing and recording transactions between different traceability units are directly covered by EPCIS Events. More detailed information about EPCIS can be obtained from Thakur et al.(2011) [16].

**Fig 1:**
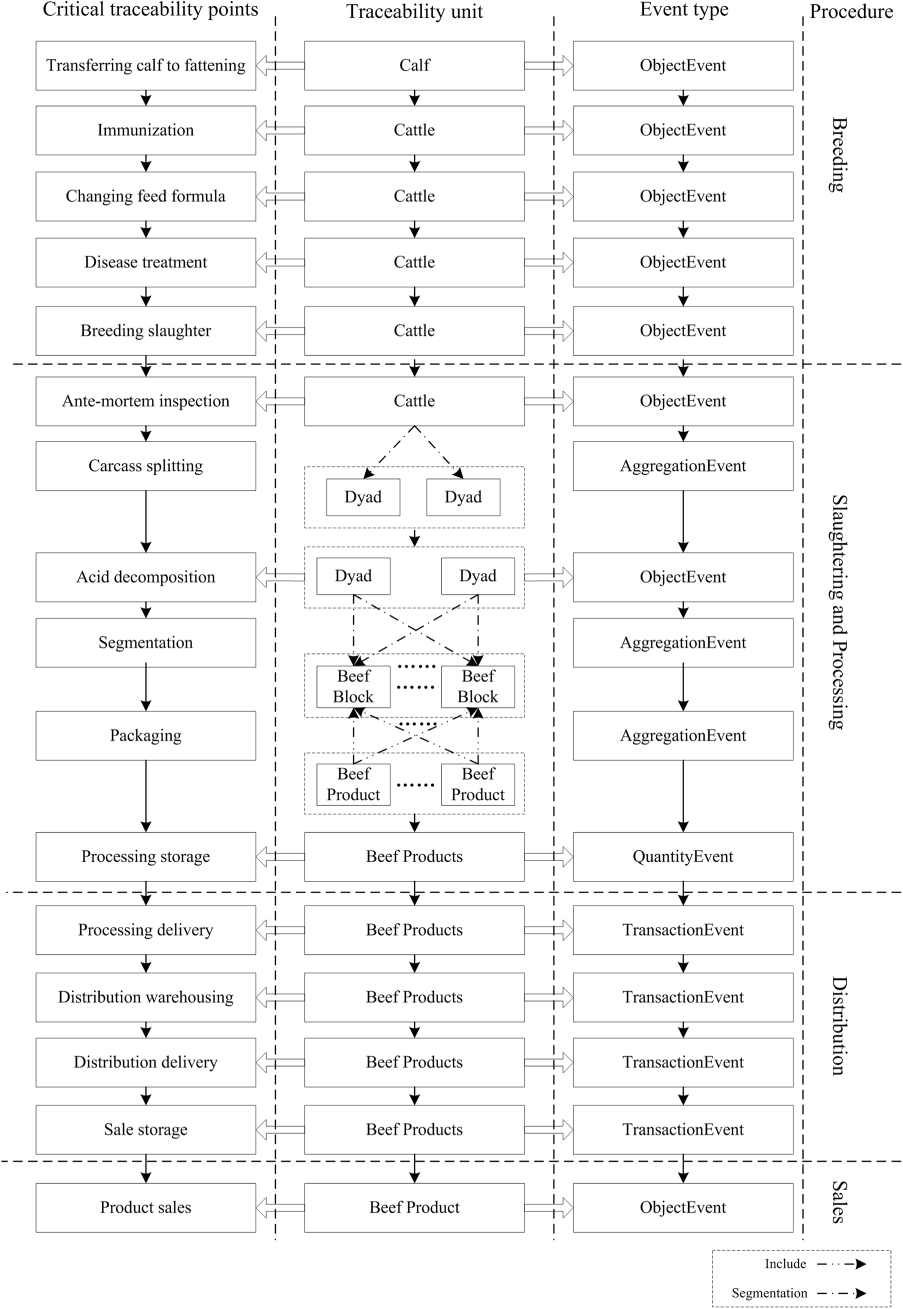
Transformation process of traceability unit and relationship between critical traceability point, event type and traceability unit.

Throughout the beef supply chain, business management systems collect and manage the product information, and convert critical production events and information into EPCIS events and external traceability information at critical traceability points. The EPCIS events and external traceability information are stored in the EPCIS server to allow the supply chain traceability of beef products. According to the beef supply chain traceability unit model and EPCIS standards, the critical traceability point, EPCIS events and traceability information of beef supply chain are summarized, and listed in Table 2. Fig 1 illustrated the transformation process of traceability unit and relationship between critical traceability point, event type and traceability unit. The business processes as follows.

**Table 2:** The critical traceability points, events and traceability information of beef supply chain.

Critical traceability points	Event type	Operation	Traceability information and comment
Transferring calf to fattening	ObjectEvent	ADD	Breed, age, size, weight, health status, date, breeding base name.
Immunization	ObjectEvent	ADD	Immunization method, medicine, dosage, operator, time.
Changing feed formula	ObjectEvent	ADD	Formula, additive content, operator, rearing stage.
Disease treatment	ObjectEvent	ADD	Disease name, treatment method, medicine, dosage, operator, time.
Breeding slaughter	ObjectEvent	ADD	Size, weight, price, health status, date.
Ante-mortem inspection	ObjectEvent	ADD	Size, weight, health status, origin, quarantine certificate of origin, operator, time.
Carcass splitting	AggregationEvent	ADD	The traceability objects transition from a cow to dyad.
Acid decomposition	ObjectEvent	ADD	Temperature, humidity, microbiological environment, start time, end time, operator.
Segmentation	AggregationEvent	ADD	The traceability objects transition from a dyad to vacuum-packed beef
Packaging	AggregationEvent	ADD	The traceability objects transition from vacuum-packed beef products into packaged beef products.
Processing storage	QuantityEvent	ADD	The quantity element’s value is the number of similar beef products.
Processing delivery	TransactionEvent	OBSERVE	Library location, batch, time, operator.
Distribution warehousing	TransactionEvent	OBSERVE	Storage location, time, operator.
Distribution delivery	TransactionEvent	OBSERVE	Library location, batch, time, operator.
Sale storage	TransactionEvent	OBSERVE	Storage location, time, operator.
Product sales	ObjectEvent	OBSERVE	Time of sale, price, cashier.

When a calf was transferred to fattening, an ear tag with a unique identification code was assigned to each calf. In the breeding process, the information related to the calf which included feeding, immunization, disease treatment, breeding slaughter, were identified by the identification code and recorded in the business database.

During the slaughter and processing stage, the operator read the identification code in the ear tag using an RFID reader, and stored the information related to the cattle in the business database after ante-mortem inspection. In the slaughter process, the information related to bloodletting, peeling, disembowelment was recorded in the business database. When the cattle carcass was segmented into two halves, the operator added one digit (1 or 2) after the ear tag code as the identification code for each dyad before writing the new code to an RFID tag and pasting the RFID tag on the dyad. The information about dyad and acid decomposition treatment was identified by the new identification code and recorded in the business database. After the acid decomposition treatment, according to the business needs and the position of the cattle, the dyads were segmented into different beef products, and the beef products were vacuum-packed. After vacuum packing, two digits were added after the dyad identification code as the identification code for the beef product and barcodes were printed on the vacuum packing boxes. In the product packaging process, the vacuum-packed beef products were packed in different packing boxes. According to the EPC standard, the operator assigned a unique SGTIN to each packed beef product and a barcode was printed on the packing box before writing the SGTIN to the RFID tag and pasting it on the packing box. The information about packed beef product was identified by the assigned SGTIN code and recorded in the business database. Finally, packaged beef products were transported to the factory warehouse, and the operator recorded the storage information in the business database.

The distribution process included processing delivery, distribution warehousing, distribution delivery and sale storage. When products leaved the storage depot, the operator read the identification code from the RFID tag on the beef product using an RFID reader and recorded the product delivery information in business database. When the beef products were transported to distribution center, the operator read the identification code from the RFID tag of the beef product using an RFID reader and recorded the warehousing information in the business database of the distribution company. When products leaved distribution center, the operator read the identification code from the RFID tag on the beef product using an RFID reader and recorded the distribution delivery information in business database. During the transport process, the transport company assigned a unique SSCC(Serial Shipping Container Code) to container and recorded the information for the waybill, driver, and vehicle in the business database of the distribution company. When the beef products were transported to sales outlets, the operator read the identification code from the RFID tag of the beef product using an RFID reader and recorded the information related to product storage in the business database of the sales outlet.

When the beef product was sold, the cashier read the information related to the beef product purchased by consumer using a point of sales (POS) machine and settled the accounts for the beef product.

## System Design

**Fig 2:**
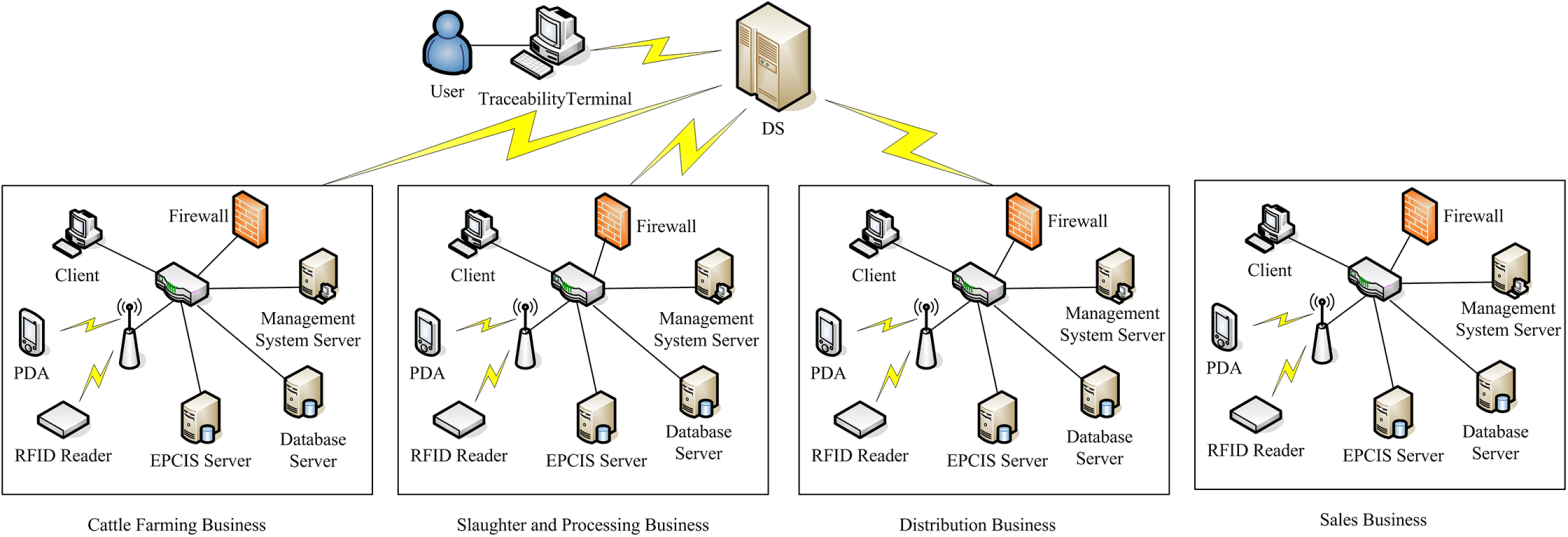
Network architecture of the traceability system.

**Fig 3:**
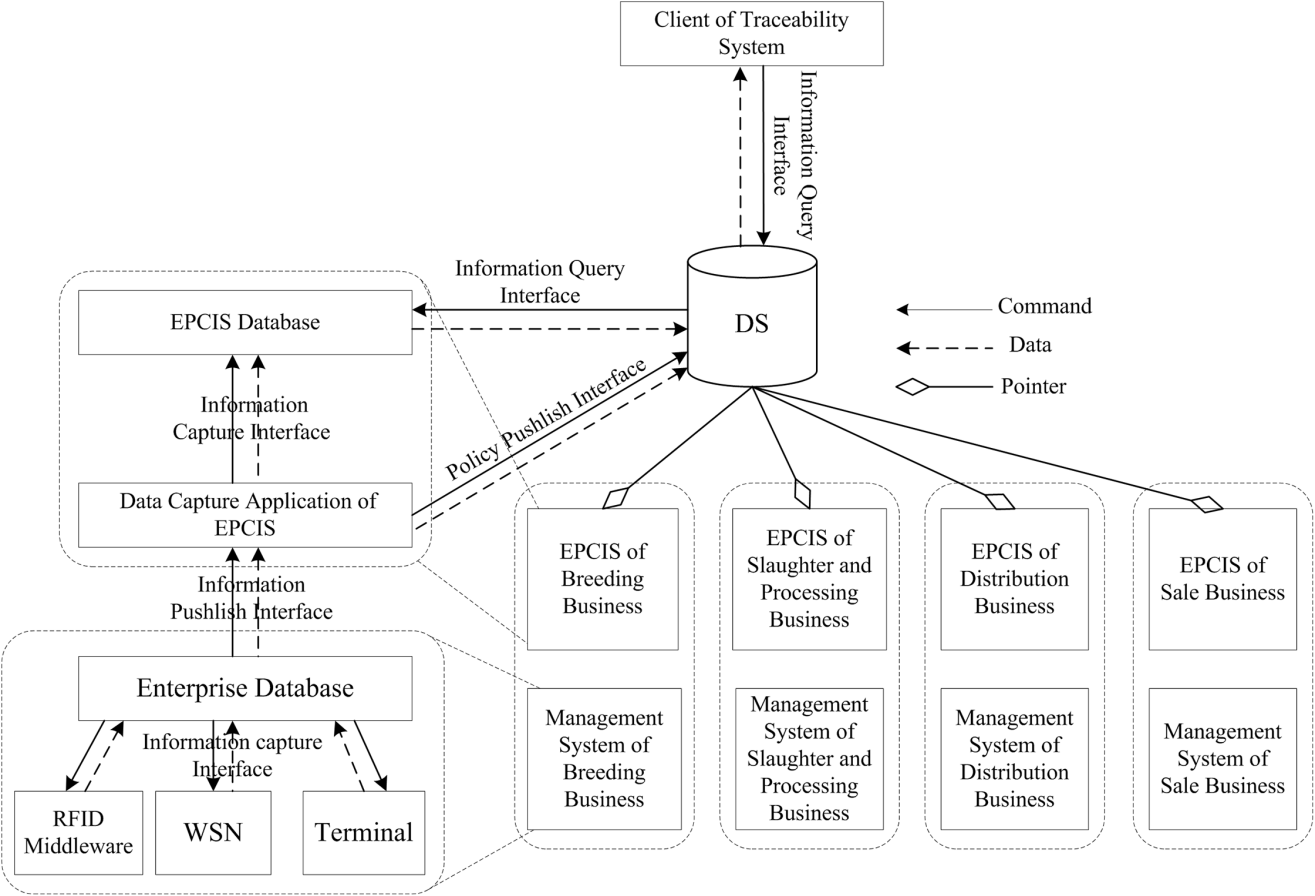
Software framework and interface of traceability system.

The traceability system proposed in this study provides an integrated framework for cattle/beef supply chain identification and traceability from cattle breeding to the dining table. The network hardware architecture of the traceability system was shown in Fig 2. The software framework and interface of the traceability system were shown in Fig 3.

### 4.1 Network architecture

Similar to most system architectures based on the EPCglobal network framework, the traceability system adopts a four-layer architecture, which comprises a perception layer, server layer, look-up server layer, and client layer.

The perception layer includes wireless sensor nodes that are designed specifically for the traceability system and RFID module. The wireless sensor nodes are managed by a self-organized and self-configured Zigbee network. The wireless sensor nodes are designed for monitoring environmental parameters related to breeding, slaughter, processing, transport, and storage, such as temperature, humidity, illumination, and the ammonia content of the air. The data are treated as a Zigbee payload and they are encapsulated in packets, which are transmitted by multi-hop via the wireless sensor network (WSN) to the wireless sensor network gateway. The gateway server collects the data in the packets from the sensor nodes and sends them to the business database server via the Internet or 3G/4G network. The RFID module with the RFID reader, writer, ear tags and tags with protective layer was used to identify the traceability unit, operator, and other objects. The RFID readers and writers are integrated in the wireless local area network (WLAN) of the beef supply chain businesses.

The server layer comprises the business management system servers, business database servers, and EPCIS servers. The cattle breeding companies, slaughter and processing businesses, and sales outlets have their own private business management system servers, business database servers, and EPCIS servers. Each business’s private servers are organized via a local area network (LAN) or wide area network (WAN), which are protected by firewalls and connected to the Internet. The server layer provides production information management, data storage, and access, as well as traceability information retrieval services.

The discovery service (DS) server is an important component of look-up server layer. The DS servers are organized via a distributed network. DS is a dynamic real-time pointer that points to the EPCIS servers which hold the events and traceability information for the EPC.

The client layer mainly provides a user visualization environment and a graphical user interface (GUI) for end users to manage business data, or to trace and track beef product information. The client layer comprises a personal digital assistant, smartphone, personal computer (PC), and terminal for the traceability of beef products, which are connected to the beef product supply chain traceability system via WLAN, LAN, WAN, 3G, or the internet.

### 4.2 Software framework and interface

The software framework and interface of the traceability system were shown in Fig 3, which comprise the DS, EPCIS, business management system, business database, data capture module, and main interface. In the beef product supply chain, each business has its own private management system and EPCIS. The business management system is responsible for production information capture and management, and the EPCIS is used to store EPCIS events and traceability information for cattle/beef products. The DS is the core component of the gapless traceability system for the beef supply chain, which provides query services for item-level information related to cattle/beef products, and it can transform a traceability identification code into a URL of the EPCIS that stores the information related to the identification code. In this study, a distributed hash table (DHT) was used to develop the distributed DS and a secure access mechanism provided by Shi et al. (2012) was employed in the design of the DS[45].

#### 4.2.1 Traceability information acquisition procedure

In the cattle/beef supply chain, each business management system captures product information and data using RFID middleware, WSN, and information input modules, thereby managing the product information and business process. At the critical traceability points, the business management system converts the internal production information into EPCIS events and traceability information for cattle/beef products, which are sent to the private EPCISs owned by businesses via an information capture interface. After receiving the information, the EPCIS stores the information and sends a security access policy that contains the identification code if it is a new one. The DS updates its security assess policy when it receive new ones.

#### 4.2.2 Traceability information query procedure

According to the overall design of the traceability system, The DS is the core component that manages and maintains the security access policy for cattle/beef identification codes. During the traceability information query procedure, there are several important steps, as follows.

Step 1: The consumer inputs the EPC of a beef product using a barcode scanner or keyboard and initiates the traceability information query process. Next, the client sends the identification code and the information query command to DS. When the identification code and command are received, the DS looks up its security access policy database and makes a decision about whether the client has access rights. If the client has access rights, the DS relays the identification code and information query command to EPCISs that contain the traceability information related to the identification code, otherwise the request is rejected.

Step 2: After receiving the identification code and command, the EPCIS looks up its database using the identification code and sends the query result to the DS.

Step 3: After receiving the query result from the EPCISs, the DS searches the result and decides whether “AggregationEvent” type events are present. If they are, the DS resolves these events and extracts the identification code included in the “parentID” element and “childEPCs” element of the event. Next, the DS finds the identification code for the parent trace object of the current trace object, before sending the identification code and information query command to EPCISs that contain the traceability information for the parent trace object. Steps 3–4 are repeated until all of the supply chain traceability information are obtained for the beef product.

Step 4: The DS sorts the query results based on time and sends the final results to the client. The traceability information is then placed in a readable form by the client and shown to the consumer.

## Implementation of the Traceability System

Auto-identification and WSN equipments were important for automatically capturing traceability information. In order to obtain more accurate information of the production environment, a compound sensor integrated temperature, humidity and illumination was developed. In this compound sensor, temperature and humidity sensors were installed in a shutter which could avoid direct sunlight, and an illumination sensor was installed at the top of the shutter. The environment parameters were captured every 10 minutes, thereby, less data traffic was suitable for remote data transmission by 3G network. GPRS-RTU(General Packet Radio Service and Remote Terminal Unit, Comway Electronic Corp, WRC-6100) was adopted for remote data transmission in this study.

Three type of RFID tags were used in the cattle beef supply chain traceability system: ear tag, UHF (Ultra High Frequency) RFID tag and self adhesive RFID label. Ear tag was applied to identify one cattle from breeding to slaughtering. The Ear tag provided LF (Low Frequency) band of 125 KHz, and had a storage capacity of 512bits and reading distance of 2–200cm. An ALIEN H3 chip was encapsulated in PPS (Phenylenesulfide) material protection layer to formulate the RFID tag, which was applied to identify beef in the slaughter and processing stage. The RFID tag had a worldwide operation in the UHF bands (860–960 MHz) and met Electronic Product Code (EPC) global Gen 2 and ISO/IEC 18000-6C specifications. Self adhesive RFID labels were pasted on the packing boxes of beef products after traceability barcodes were printed on and the SGTINs were written. It had lower prices, and a maximum long reading distance of 3 m.

**Fig 4:**
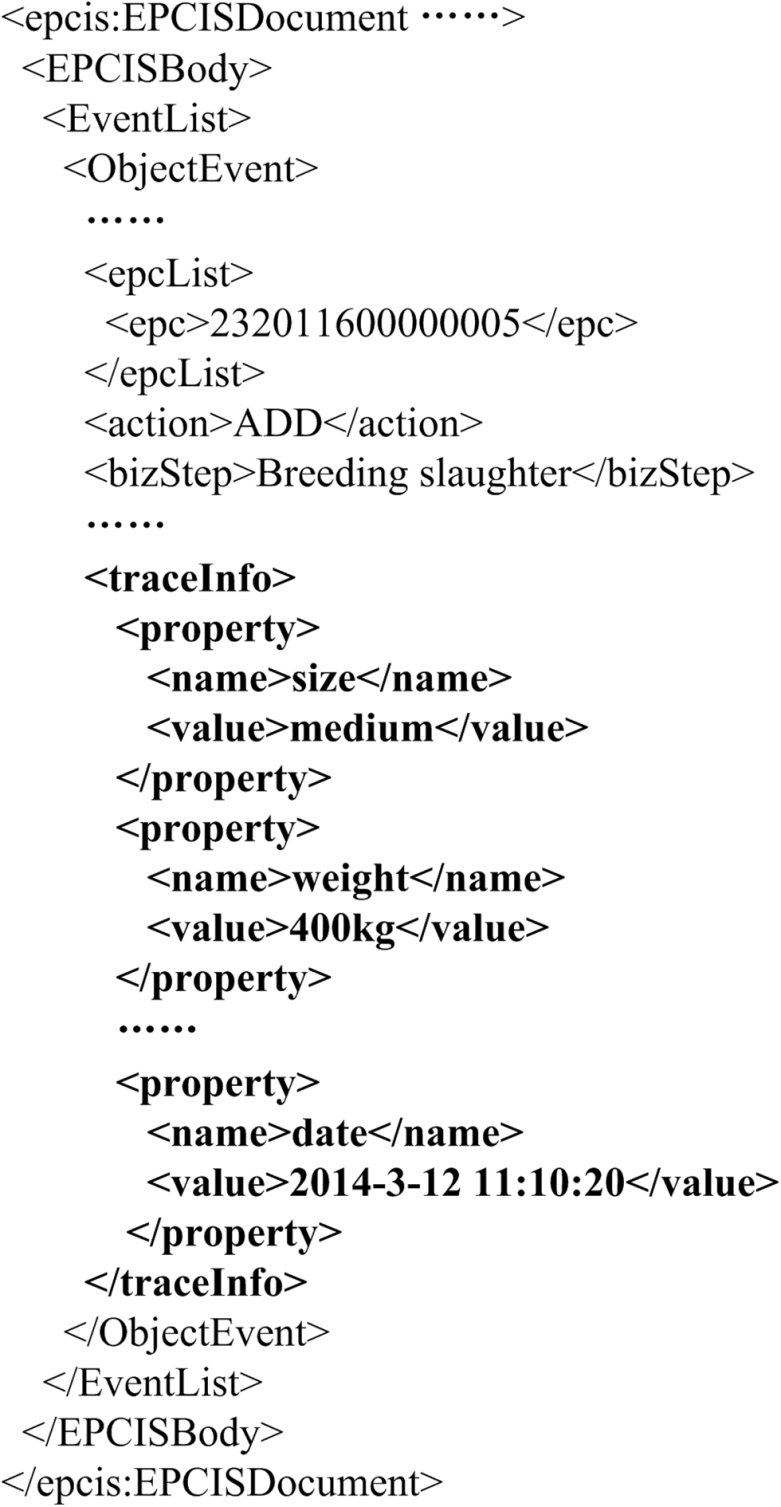
A sample of the EPCIS capture document (XML).

The architecture of the traceability system was divided into a set of logical components, as shown in Fig 3. Fosstrak [46] was used to develop the EPCIS, where its database table structure, data capture interface, and information query interface were extended to meet the system design requirements. In the EPCIS database, a column named “traceinfo” was added to tables named “event_objectevent” and “event_transactionevent”, which was used for storing external traceability information. AS shown in Fig 4, the XML (Extensible Markup Language) data captured by data capture interface of EPCIS was modified, where the “traceinfo” element was added to “ObjectEvent” element. The data capture interface and information query interface were extended for parsing and dealt with the modified XML data.

According to the design of the DS, DHT applications require a structured P2P (peer-to-peer) network as a substrate. FreePastry is a modular, open source implementation of the pastry p2p structured overlay network[47]. Therefore, it was used for implementing the DS in the traceability system. Based on FreePastry, the information query interface, policy publish/query interface, policy management, relay decision, and relay interface of the DS were developed.

**Fig 5:**
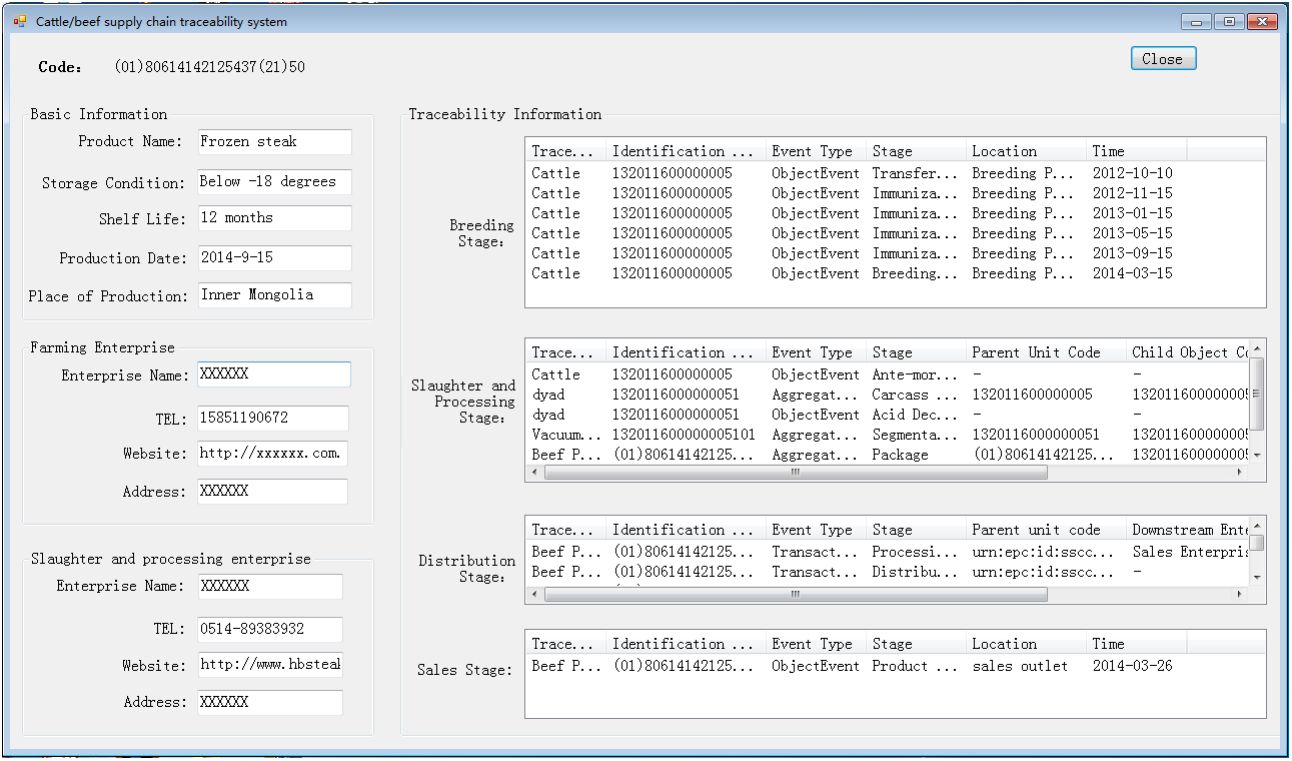
Interface of the traceability system for traceability information query.

**Fig 6:**
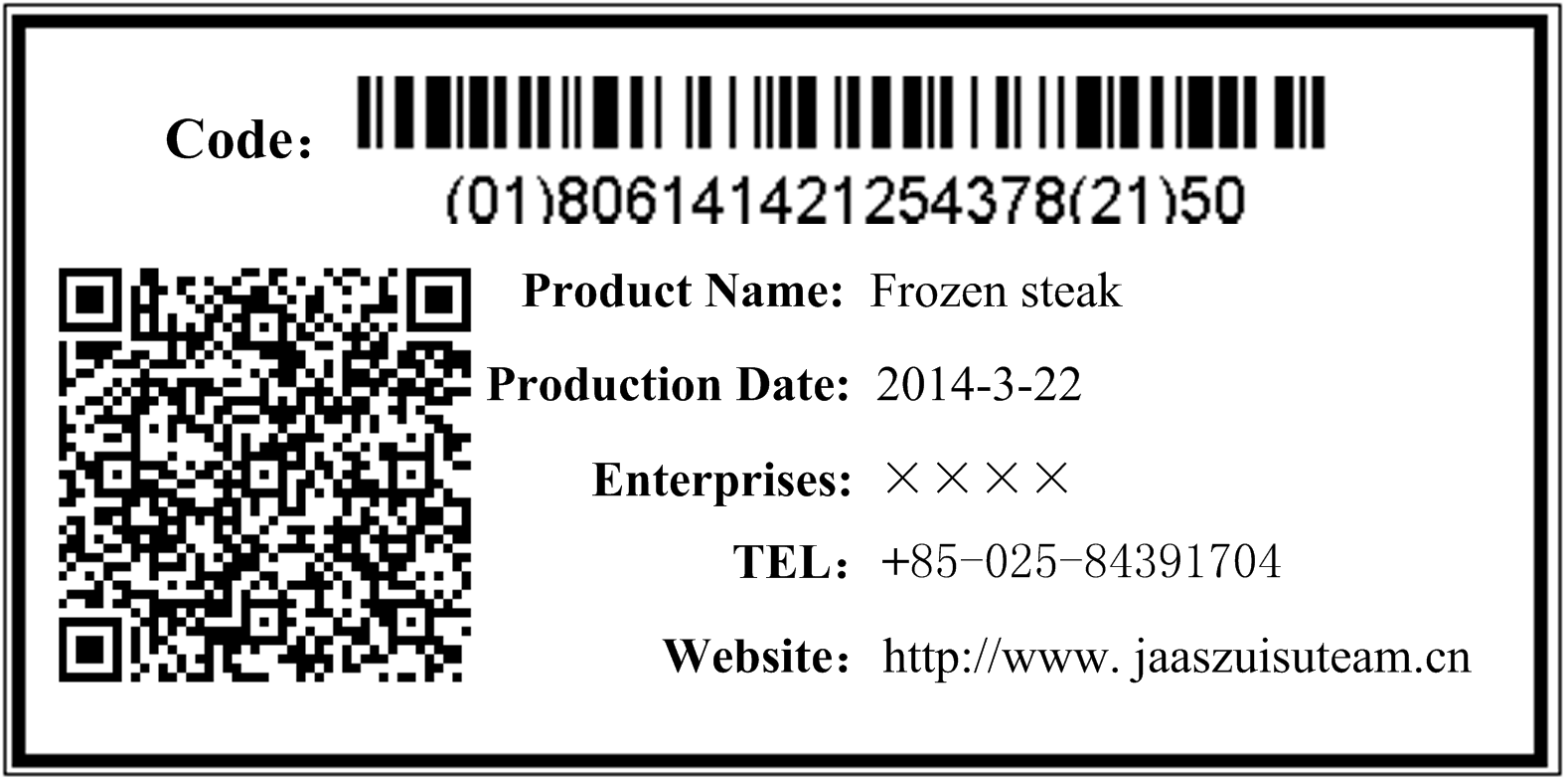
Traceability label of beef product.

The business management systems were developed using Java, JSP, Struts, etc, which include external traceability information release interface, RFID middleware, information capture interface, etc. Fosstrak ALE Middleware, Fosstrak TDT components, and barcode generation components were integrated in the system to implement the RFID tag reader and writer middleware. MySQL Enterprise Edition was used to construct the business databases. Fig 5 showed the interface of the traceability system used for traceability information queries and Fig 6 showed a traceability label printed on beef product packaging.

## System Evaluation and Discussion

### 6.1 System test

With the support of the project partners, Liangfeng cattle farm, a farm with 3000 cattle, located in south eastern China, Zhangjiagang city, and SG, a chain supermarket with advanced logistics distribution system located in Nanjing city were selected for the system testing and evaluation. In order to form a complete supply chain, a slaughter and processing enterprises was selected. For shorten the test time, five fattening calves were selected for trial, and the prior information of transferring calf to fattening, immunization, changing feed formula and disease treatment were input based on historical records. The information of the five calves was captured by the traceability system until they were sent to the slaughterhouse. During the slaughter and processing phases, the traceability system was adopted for the five calves. Finally, the beef products of the five calves were sent to SG supermarket and sold in a community shop in Nanjing city. In the community shop, a computer with touch screen was used for consumers to apply the traceability system.

The DS server was installed in the data center of our institute, which was used for look-up service. In order to simplify the system configuration, the EPCIS, business management system were also installed independently in the data center of our institute. The client, WSN and RFID reader/writer were installed respectively in the enterprises of the case study.

During the trial process, system users were encouraged to take part in the system trial and provide their feedback and comments. After the testing, questionnaires were carried out to system users and consumers. The major questions of questionnaires are listed in Table 3 and Table 4.

**Table 3:** Major questions of system user questionnaire.

No	Question	Answer1	Answer2	Answer3	Answer4
Q1	System usability	Poor	Acceptable	Good	Excellent
Q2	System stability	Poor	Acceptable	Good	Excellent
Q3	System scalability	Poor	Acceptable	Good	Excellent
Q4	User interface	Poor	Acceptable	Good	Excellent
Q5	Ease of use	Difficult	General	Ease	Very Ease
Q6	Cost	High	Acceptable	Low	Can be ignored

**Table 4:** Major questions of consumer questionnaire.

No	Question	Answer1	Answer2	Answer3	Answer4
Q1	Information is complete?	No	Lack of key information	Complete	Very complete
Q2	Credibility of the information?	No	Partially	Trust	Completely trust
Q3	Ease of use of the system?	Difficult	General	Ease	Very Ease

### 6.2 Results and discussion

**Fig 7:**
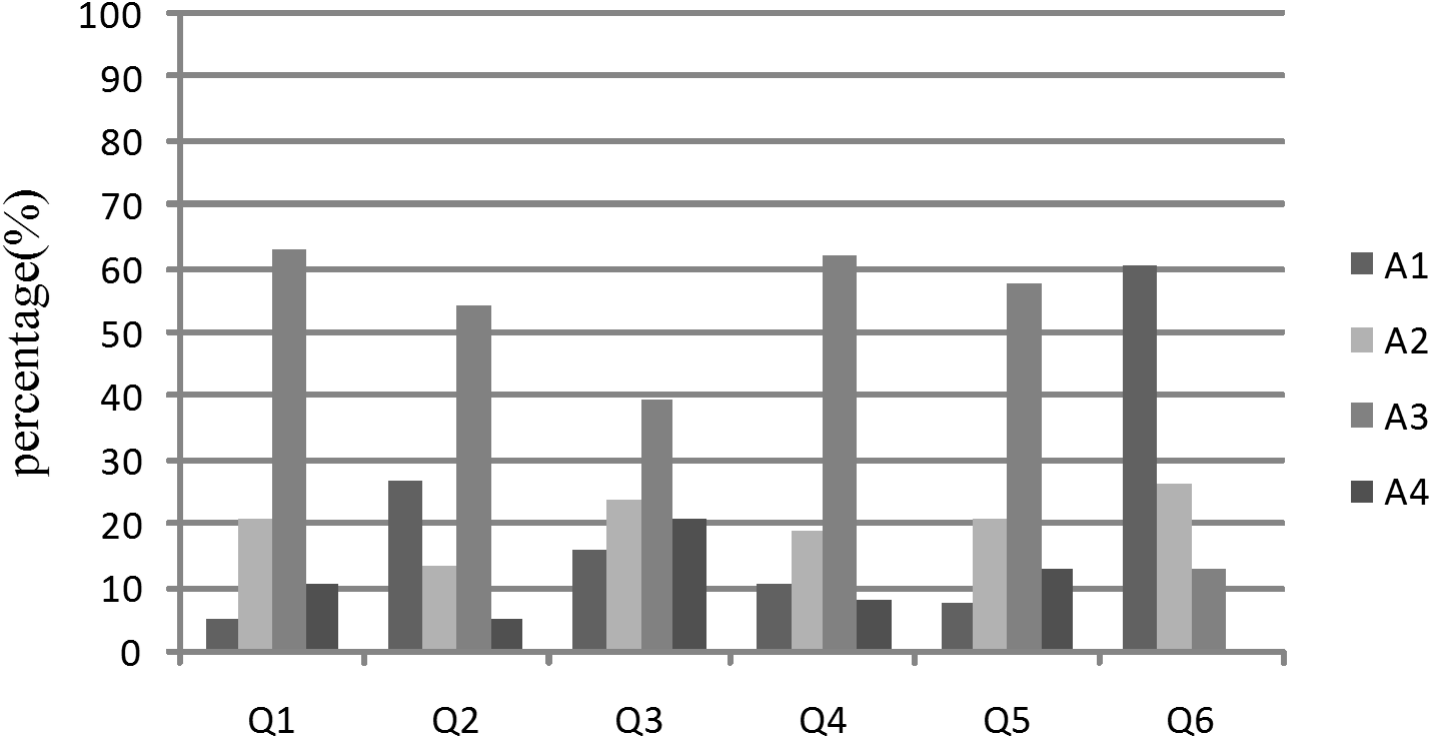
Statistical analysis result of questionnaires obtained from system users.

**Fig 8:**
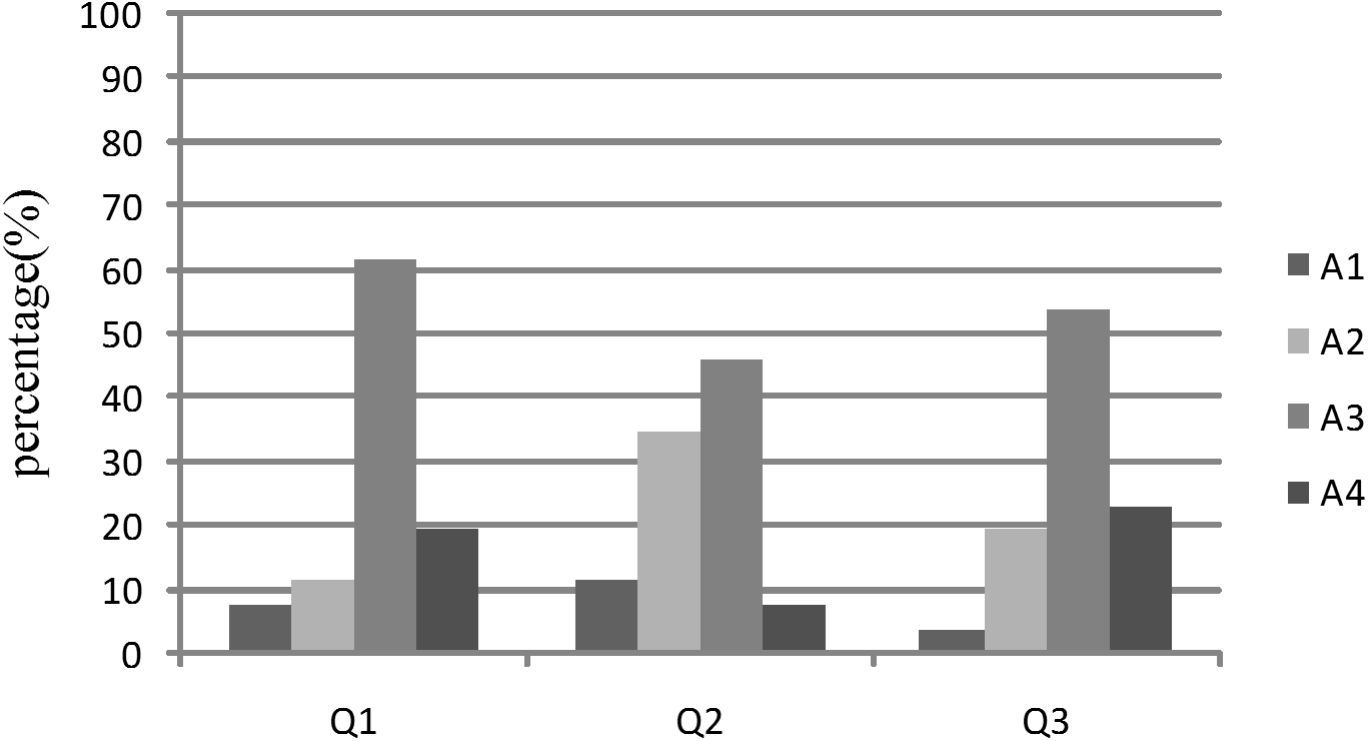
Statistical analysis result of questionnaires obtained from consumers.

After the completion of the investigation, 38 questionnaires of system users and 26 questionnaires of consumers were received. The statistical analysis results were shown in Fig 7 and Fig 8.

#### 6.2.1 Evaluation by the system users

As shown in Fig 7, the results of the first three questions showed that the system could realize the traceability of cattle/beef supply chain, automatically acquire, transmit and processing data, and has good stability and scalability. However, some users considered that the system stability is poor (Q2). After field investigation and testing, we found that the stability of the WSN was affected by surrounding environment. Thereby, WSN was instead of wired sensor network in the complex environment.

In terms of system user experience, about 70% of the system users considered that the system has a user-friendly interface and users can quickly learn how to use it based on the user’s manual (Q4 and Q5). The inputting of traceability information was perceived as complex and time-consuming for ordinary users. In this system, WSN and RFID were used to collect the information, which improved the level of automation. Furthermore, menu and code input methods were provided to reduce the information input workload. Moreover, at critical traceability points, the internal traceability information can be converted automatically into external traceability information by the business management system.

The statistical analysis result showed that about 60% of the system users considered that the cost was expensive (Q6).The hardware and software are one-time investments, and RFID tags are the major consumables. The high cost of RFID tags is the major component of the operating costs. During the breeding process, ear tags are disposable products and their cost is proportional to the amount of cattle. The price of per ear tag is around 5–10 Chinese Yuan in 2015. The investment of a farm with 3000 cattle is 15000–30000 Chinese Yuan, which had not taken into account of the possible damage of RFID tags. During the slaughter and processing stage, the RFID tags with protective layer can be recycled, only the RFID tags pasted onto beef products entered the downstream business process. Hence, in order to reduce the system costs, a bar code label with RFID tag has been used for storing and printing traceability code of beef product. The price of this type label/tag is around 0.45 Chinese Yuan. The distribution and sales businesses do not incur any costs in terms of RFID tags. However, the traceability system can increase consumer’s confidence, which should increase companies´ sales and benefits[39].

#### 6.2.2 Evaluation by the consumers

From the evaluation and feedback (Fig 8), we can conclude that the traceability system can provides gapless and detailed traceability information to consumers and it is relatively easy to use. In this system, the EPCISs owned by businesses were linked by the DS, which facilitated the gapless connection of traceability information. During the traceability unit segmentation and packaging process, AggregationEvent was used to establish the relationships between parent and child traceability units. The gapless traceability of beef products throughout the supply chain was facilitated based on this relationship when beef products were traced. However, there is mistrust about the authenticity of the traceability information, and no one trust completely. Therefore, there is still a lot of work to do, for example, expanding the application scope of the system, introduction of third-party authentication mechanisms, etc.

## Conclusions

This paper proposed a beef supply chain traceability system based on RFID technology and EPCglobal network. In the system, the animal ear tag codes are used for identification of traceability units. In the model of traceability information, the EPCIS events and traceability information related to cattle/beef are designed in detail, and AggregationEvent is used to establish the relationships between parent and child traceability units when the traceability units are segmented or packaged. The EPCIS was developed based on Fosstrak, where its database table structure, data capture interface, and information query interface were extended. The DS was implemented based on FreePastry, which integrated all the EPCISs owned by enterprises of cattle/beef supply chain.

System trial results showed that the system could realize the gapless traceability of cattle/beef supply chain, and has good stability and scalability. Most system users and consumers considered that the system has a user-friendly interface and is relatively easy to use. However, there existed some barriers for system adoption: the instability of WSN, the high implementation cost, and the mistrust of the authenticity of traceability information. The first problem can be solved by using wired sensor network in the complex environment. With the development of technology, the cost of RFID tags is getting lower and lower. Moreover, a bar code label with RFID tag used for storing and printing traceability code, could reduce the implementation cost. The authenticity issue of traceability information was a concern. Therefore, the application scope of the system should be expanded or a third-party authentication mechanism of traceability information is introduced, which are our next working directions.

## References

[1] Qi L, Zhang J, Xu M, Fu ZT, Chen W, Zhang XS. Developing WSN-based traceability system for recirculation aquaculture. Mathematical and Computer Modelling, 2011, 53(11–12):2162–2172.

[2] Comba L, Belforte G, Dabbene F, Gay P. Methods for traceability in food production processes involving bulk products. Biosystems Engineering, 2013, 116(1): 51–63.

[3] Dabbene F, Gay P, Tortia C. Traceability issues in food supply chain management: A review. Biosystems Engineering, 2014, 120: 65–80.

[4] Moe T. Perspectives on traceability in food manufacture. Trends in Food Science & Technology, 1998, 9(5): 211–214.

[5] Banterle A, Stranieri S. The consequences of voluntary traceability system for supply chain relationships. An application of transaction cost economics. Food Policy, 2008, 33(6): 560–569.

[6] Engelseth P. Food product traceability and supply network integration. Journal of Business & Industrial Marketing, 2009, 24(5/6): 421–430.

[7] Canavari M, Centonze R, Hingley M, Spadoni R. Traceability as part of competitive strategy in the fruit supply chain. British Food Journal, 2010,112(2):171–184.

[8] Frederiksen MT, Bremner A. Fresh fish distribution chains: An analysis of three Danish and three Australian chains. Food Australia, 2001, 54(4):117–123.

[9] Bertolini M, Bevilacqua M, Massini R. FMECA approach to product traceability in the food industry. Food Control, 2006, 17(2): 137–145.

[10] Donnelly KAM, Karlsen KM, Dreyer B. A simulated recall study in five major food sectors. British Food Journal, 2012, 114(7):1016–1031.

[11] Främling K, Ala-Risku T, Kärkkäinen M, Holmström J. Agent-based model for managing composite product information. Computers in Industry, 2006, 57(1):72–81.

[12] Bechini A, Cimino MGCA, Marcelloni F, Tomasi A. Patterns and technologies for enabling supply chain traceability through collaborative e-business. Information and Software Technology, 2008, 50(4): 342–359.

[13] Li M, Qian JP, Yang XT, Sun CH, Ji ZT. A PDA-based record-keeping and decision-support system for traceability in cucumber production. Computers and Electronics in Agriculture, 2010, 70(1):69–77.

[14] Karlsen KM, Olsen P. Validity of method for analysing critical traceability points. Food Control, 2011, 22(8):1209–1215.

[15] Donnelly KA, Karlsen KM, Olsen P. The importance of transformations for traceability—A case study of lamb and lamb products. Meat Sci, 2009, 83(1):68–73. doi: 10.1016/j.meatsci.2009.04.006 20416638 10.1016/j.meatsci.2009.04.006

[16] Thakur M, Sørensen CF, Bjørnson FO, Forås E, Hurburgh CR. Managing food traceability information using EPCIS framework. Journal of Food Engineering, 2011, 103(4):417–433.

[17] Pizzuti T, Mirabelli G, Sanz-Bobi MA, Goméz-Gonzaléz F. Food Track & Trace ontology for helping the food traceability control. Journal of Food Engineering,2014,120:17–30.

[18] Pizzuti T, Mirabelli G. The Global Track&Trace System for food: General framework and functioning principles. Journal of Food Engineering,2015,159:16–35.

[19] Thakur M, Hurburgh CR. Framework for implementing traceability system in the bulk grain supply chain. Journal of Food Engineering, 2009, 95(4): 617–626.

[20] Hu JY, Zhang X, Moga LM, Neculita M. Modeling and implementation of the vegetable supply chain traceability system. Food Control, 2013, 30(1): 341–353.

[21] Musa A, Gunasekaran A, Yusuf Y. Supply chain product visibility: Methods, systems and impacts. Expert Systems with Applications, 2014, 41(1):176–194.

[22] Costa C, Antonucci F, Pallottino F, Aguzzi J, Sarri D, Menesatti P.A review on agri-food supply chain traceability by means of RFID technology.Food and Bioprocess Technology 2,2013,6(2):353–366.

[23] Hong I, Dang J, Tsai Y, Liu C, Lee W, Wang M, et al An RFID application in the food supply chain: a case study of convenience stores in Taiwan. Journal of Food Engineering, 2011,106(2),119–126.

[24] Jones P, Clarke-Hill C, Shears P, Comfort D, Hillier D. Radio frequency identification in the UK: opportunities and challenges. International Journal of Retail & Distribution Management, 2004,32(3): 164–171.

[25] Koutsoumanis K, Taoukis PS, Nychas GJE. Development of a safety monitoring and assurance system for chilled food products. International Journal of Food Microbiology, 100(1–3), 2005:253–260. 15854710 10.1016/j.ijfoodmicro.2004.10.024

[26] Twist DC.The impact of radio frequency identification on supply chain facilities. Journal of Facilities Management,2005,3(3):226–239.

[27] Attaran M. RFID: an enabler of supply chain operations. Supply Chain Management-an International Journal,2007,12(4):249–257.

[28] Abad E, Palacio F, Nuin M, Zárate A, González de Juarros A, Gómez JM, et al RFID smart tag for traceability and cold chain monitoring of foods: Demonstration in an intercontinental fresh fish logistic chain.Journal of Food Engineering,2009,93(4):394–399.

[29] Qian JP, Yang XT, Wu XM, Zhao L, Fan BL, Xing B.A traceability system incorporating 2D barcode and RFID technology for wheat flour mills.Computers and Electronics in Agriculture,2012,89:76–85.

[30] Piramuthu S, Farahani P, Grunow M. RFID-generated traceability for contaminated product recall in perishable food supply networks.European Journal of Operational Research,2013,225(2):253–262.

[31] Barge P, Gay P, Merlino V, Tortia C. Item-level Radio-Frequency IDentification for the traceability of food products: Application on a dairy product. Journal of Food Engineering,2014,125:119–130.

[32] Jakkhupan W, Arch-int S, Li YF. Business process analysis and simulation for the RFID and EPCglobal Network enabled supply chain: A proof-of-concept approach. Journal of Network and Computer Applications, 2011, 34(3):949–957.

[33] Manzanares-Lopez P, Muñoz-Gea JP, Malgosa-Sanahuja J, Sanchez-Aarnoutse JC. An efficient distributed discovery service for EPCglobal network in nested package scenarios. Journal of Network and Computer Applications, 2011, 34(3): 925–937.

[34] Muñoz-Gea JP, Malgosa-Sanahuja J, Manzanares-Lopez P, Sanchez-Aarnoutse JC. Implementation of traceability using a distributed RFID-based mechanism. Computers in Industry, 2010, 61(5): 480–496.

[35] Nam T, Yeom K. Business-aware framework for supporting RFID-enabled applications in EPC Network. Journal of Network and Computer Applications, 2011, 34(3):958–971.

[36] Ko J, Kwak C, Cho Y, Kim CO. Adaptive product tracking in RFID-enabled large-scale supply chain. Expert Systems with Applications, 2011, 38(3):1583–1590.

[37] Kang YS, Lee YH. Development of generic RFID traceability services. Computers in Industry, 2013, 64(5):609–623.

[38] Mainetti L, Patrono L, Stefanizzi ML, Vergallo R. An innovative and low-cost gapless traceability system of fresh vegetable products using RF technologies and EPCglobal standard. Computers and Electronics in Agriculture, 2013, 98:146–157.

[39] Parreño-Marchante A, Alvarez-Melcon A, Trebar M, Filippin P. Advanced traceability system in aquaculture supply chain. Journal of Food Engineering, 2014, 122:99–109.

[40] Karlsen KM, Dreyer B, Olsen P, Elvevoll EO. Literature review: Does a common theoretical framework to implement food traceability exist? Food Control, 2013, 32(2):409–417.

[41] Feng JY, Fu ZT, Wang ZQ, Xu M, Zhang XS.Development and evaluation on a RFID-based traceability system for cattle/beef quality safety in China.Food Control,2013,31(2):314–325.

[42] MOA Ministry of Agriculture of the People’s Republic of China, 2006. Regulation on administration of livestock and poultry identification and the breeding files: No.67. Ministry of Agriculture of the People’s Republic of China (10/06/2009). Available: http://www.moa.gov.cn/zwllm/tzgg/bl/200606/t20060628_638621.htm.

[43] NBSC National Bureau of Statistics of China, 2006. Codes of administrative regions of the People’s Republic of China (02/07/2009). Available: http://www.stats.gov.cn/tjbz/xzqhdm/t20070411_402397928.htm.

[44] EPC Tag Data Standard, 2014. The EPC Tag Data Standard defines the Electronic Product Code, Version 1.8^TM^ Available: http://www.gs1.org/gsmp/kc/epcglobal/tds.

[45] Shi J, Li YJ, He W, Sim D. SecTTS: A secure track & trace system for RFID-enabled supply chains. Computers in Industry,2012,63(6):574–585.

[46] Fosstrak, Available: http://www.fosstrak.org.

[47] FreePastry, Available: http://www.freepastry.org/FreePastry.

